# Optimised comorbidity indices can reflect patient performance status in register based studies of prostate cancer

**DOI:** 10.1038/s41598-025-32190-9

**Published:** 2025-12-26

**Authors:** Marcus Westerberg, Hans Garmo, Jesper Bonnedahl, Marie Hjälm Eriksson, David Robinson, Pär Stattin, Rolf Gedeborg

**Affiliations:** 1 Department of Surgical Sciences, Uppsala University, Uppsala, Sweden; 2 Department of Urology, Institute of Diagnostics and Intervention, Umeå University, Umeå, Sweden; 3 Department of Surgery and Oncology, Capio S:T Görans Hospital, Stockholm, Sweden; 4 Department of Urology, Ryhov Hospital, Jönköping, Sweden; 5 Regional Cancer Center Midsweden, Uppsala University Hospital, 752 37 Uppsala, Sweden

**Keywords:** Comorbidity, ECOG, Performance status, Prostate cancer, Cancer epidemiology, Prostate cancer, Cancer, Prostate

## Abstract

Eastern Cooperative Oncology Group Performance Status (ECOG-PS) is commonly used in cancer trials to select a study population with good performance, but ECOG-PS is rarely available in health-care registers. We assessed if patient age and comorbidity indices can substitute ECOG-PS when selecting men in register-based studies of advanced prostate cancer. ECOG-PS data for 3966 men on androgen deprivation therapy for prostate cancer were retrieved from Prostate Cancer data Base Sweden. Logistic regression models were used to discriminate between ECOG-PS 0–1 versus 2–4 based on age, Charlson comorbidity index (CCI), a novel Multidimensional Diagnosis-based Comorbidity Index (MDCI) based on ICD codes, and a Drug Comorbidity Index (DCI) based on filled prescriptions. The model based on age, MDCI, and DCI provided the best discrimination (AUC = 0.82; 95% CI 0.81–0.84). In a hypothetical cohort of 1000 men where 750 men had ECOG-PS 0–1, 600 men would be included when excluding those with high risk of ECOG-PS 2–4 using this model and 60 of these would have ECOG 2–4 instead of 250 men if all 1000 men had been included. Age and two new comorbidity indices can with reasonable precision substitute ECOG-PS and help identify subsets of study populations likely to have favourable ECOG-PS.

**Supplementary Information:**

The online version contains supplementary material available at 10.1038/s41598-025-32190-9.

## Introduction

In oncological randomized clinical trials (RCT), inclusion is often restricted to patients with a favourable performance status since this is strongly related to tolerability of therapies and survival^1–7^. The Eastern Cooperative Oncology Group performance status (ECOG-PS) scale is a commonly used performance status that describes a patient’s level of independence from care takers, daily activity, and physical ability. However, in administrative health care registers and clinical cancer registers ECOG-PS is often missing or has low coverage and completeness.

Before implementing novel therapies from study populations in a RCTs to patients in clinical practise who often have a larger burden of comorbidities and lower functional status it is useful to emulate a target trial with restriction to patients with favourable ECOG-PS, and benchmark the result against the existing RCT and then widen the use further^8–11^. A potential remedy for the lack of data on ECOG-PS is to use information on comorbidity from health care registers, which is expected to correlate with performance status. ECOG-PS has previously been substituted by use of the Charlson Comorbidity Index (CCI) and other information in different cancer populations^12–14^.

The aim of this study was to assess to what extent information on age and comorbidity can substitute for ECOG performance status in men with advanced prostate cancer in register-based studies, and to illustrate the utility of these data for patient selection in target trial emulations.

## Materials

We used data from Prostate Cancer data Base Sweden (PCBase) Extended Treatment and Endpoint Data (Xtend), a database linking The National Prostate Cancer Register of Sweden (NPCR) and its sub-register the Patient-overview Prostate Cancer (PPC)^15^ with several national administrative health care registers, including the Patient Register^16^ and the Prescribed Drug Register as previously described^10,17^. The PPC is a digital tool that provides the treating physician with a consolidated view of a patient’s disease and treatment history. It contains longitudinal data on treatment, lab data, imaging, and clinical assessment of progression in men with advanced Pca on androgen deprivation therapy (ADT). Information on ECOG-PS is often, but not always, recorded at each health care contact, and may sometimes be registered retrospectively based on information in medical records*.*

The Charlson Comorbidity Index (CCI) was calculated based on ICD-10 diagnoses registered in the National Patient Register up to 10 years prior to the date of each contact^18–21^. In addition to CCI we used two additional comorbidity indices that we have recently created. The Multi-dimensional Diagnosis-based Comorbidity Index (MDCI) is based on occurrence, frequency, recency, and total duration of associated hospital stays, using associated International Classification of Diseases (ICD) codes registered in the National Patient Register as related to hospitalizations or specialist outpatient visits up to 10 years prior to the date of each contact^22^. The Drug Comorbidity Index (DCI) is based on filled prescriptions in the Prescribed Drug Register up to 1 year prior to the date of the contact and has been validated also in patients with advanced prostate cancer^23,24^. The CCI, MDCI and DCI were computed based only on hospitalizations and drug fillings that occurred prior to the date of the corresponding healthcare contact.

### Inclusion criteria

All men in PCBase Xtend diagnosed with prostate cancer and registered in PPC were eligible. All unique dates of health care contacts (in-person or by phone; but not video chat, letter or unknown type of contact) between 2014-01-01 and 2020-12-31 were included if an ECOG-PS was recorded at the date of the contact. We included only ECOG-PS data that were documented on the day of the healthcare encounter, ensuring optimal reliability for benchmarking purposes. Retrospectively recorded ECOG-PS from medical charts may be prone to recall bias or misclassification, particularly when based solely on secondary information. In a sensitivity analysis, we also included retrospectively recorded ECOG-PS. Note that a man could contribute with multiple contacts with potentially different ECOG-PS, age, CCI, MDCI and DCI.

### ECOG performance status

ECOG-PS is defined on a scale 0–5 where 0 indicates that the subject is fully active and can carry on normal activities, 1 indicates that the subject cannot perform physically demanding activities but is able to carry out lighter work, 2 indicates that the subject is capable of all selfcare, active during at least 50% of the day, but unable to carry out any work activities, while 3 indicates that the subject is capable of only limited selfcare and is confined to bed or chair during more than half of the day, and 4 indicates that the subject is completely disabled, is completely dependent on assistance with daily care, and spends the entire day in a bed or chair, and 5 indicates that the subject is dead^25^. We consider ECOG-PS 0-4 in this study.

### Outcome

The primary outcome was defined based on a dichotomization of ECOG-PS to indicate favourable versus unfavourable performance status: ECOG-PS 0–1 versus 2–4. We defined a ‘positive’ outcome as ECOG-PS 0–1. In supplementary analyses, two other dichotomizations were used: ECOG-PS = 0 versus 1–4 and ECOG-PS = 0–2 versus 3–4. We correspondingly defined a ‘positive’ outcome as ECOG-PS 0 in the first supplementary analysis and 0–2 in the second supplementary analysis.

## Methods

We developed generalized additive logistic regression models where each covariate was represented using a thin plate regression splines where knot placement is effectively automated^26,27^. Each model estimated a probability *p* for favourable performance status (ECOG-PS 0–1 in the primary analysis). Model performance was evaluated by computing the sensitivity and specificity at different cut-offs *c* of the predicted probability^28^.The test was positive (favourable ECOG-PS) if *p* ≥ *c*. Sensitivity was defined as the proportion of men with *p* ≥ *c* among all men with favourable ECOG-PS, and specificity was defined as the proportion of men with *p* < *c* among men with unfavourable ECOG-PS (primarily ECOG-PS 2–4). The receiver operating characteristic (ROC) curve was used to visualize the trade-off between sensitivity and specificity, and overall predictive performance was estimated by the area under the ROC curve (AUC)^28^, and positive and negative predictive values. Since the primary aim was to use the model to select a subpopulation of patients with favourable ECOG-PS, we focused on achieving high specificity. For the primary intended use, we considered an operational cutoff for the specificity of 0.75, and we additionally assessed the cutoffs 0.5, 0.9, 0.95, 0.975 and 0.99. In addition, we also computed the positive predictive value (PPV) and negative predictive value (NPV) for each cutoff based on the in-study prevalence.

The basic model included only age, to which we then added CCI, or MDCI and DCI. In a supplementary analysis, we considered additional combinations of CCI, MDCI and DCI with age.

We obtained 95% confidence intervals (CIs) via bootstrapping using the percentile method and 500 replications^29^, where men were resampled at individual level to handle the dependence between repeated measurements of ECOG-PS within an individual. The AUC was corrected for optimism using Harrell’s bias correction and the corrected CIs were computed using the location-shift method^30,31^. We computed brier scores and assessed model calibration by computing calibration curves that compare predicted probabilities with empirical proportions using the *CalibrationCurves* R-package^32^. The analysis was performed using R version 4.1.3^33^. We completed the STROBE checklist (Supplementary Table 1)^34^.

This study was approved by the Swedish Ethical Review Authority *Etikprövningsmyndigheten* (2020-03,437, 2022-05,083-02) and the need for informed consent was waived by Swedish Ethical Review Authority. All research was performed in accordance with relevant guidelines and regulations.

## Results

### Baseline characteristics

There were 11 448 men with advanced prostate cancer registered in PPC with 85 738 health care contacts during 2014–2020, out of which 54 121 (63%) also had a registered ECOG-PS score. 49 750 (92%) of these contacts were made in-person or by phone. Out of these, 26% (12 792 contacts in 3966 men) ECOG-PS registrations were made at the day of the contact and included in the study (Table 1, Supplementary Fig. 1).

**Table 1:** Baseline characteristics stratified by The Eastern Cooperative Oncology Group performance status (ECOG-PS) at the time of a health care contact for 3968 men diagnosed with advanced prostate cancer in Patient-Overview Prostate Cancer (PPC) in the National Prostate Cancer Register (NPCR) of Sweden.

	Eastern Cooperative Oncology Group performance status (ECOG-PS)									
	All		0		1		2		3–4	
N health care contacts (%)	12 792	(100)	6148	(100)	4181	(100)	1855	(100)	608	(100)
Type of health care contact										
In-person	9329	(73)	4439	(72)	3082	(74)	1387	(75)	421	(69)
By phone	3463	(27)	1709	(28)	1099	(26)	468	(25)	187	(31)
Age at assessment of ECOG-PS; years										
Median (IQR)	75 (70–81)		73 (68–78)		76 (72–82)		79 (74–85)		79 (74–84)	
< 70	2724	(21)	1744	(28)	687	(16)	225	(12)	68	(11)
70–74	2743	(21)	1594	(26)	809	(19)	260	(14)	80	(13)
75–79	3195	(25)	1519	(25)	1083	(26)	451	(24)	142	(23)
≥ 80	4130	(32)	1291	(21)	1602	(38)	919	(50)	318	(52)
Year of contact										
2014–2016	259	(2)	139	(2)	85	(2)	28	(2)	7	(1)
2017–2018	3221	(25)	1628	(26)	1069	(26)	397	(21)	127	(21)
2019–2020	9312	(73)	4381	(71)	3027	(72)	1430	(77)	474	(78)
Charlson comorbidity index										
0	5234	(41)	3078	(50)	1517	(36)	492	(27)	147	(24)
1	1736	(14)	712	(12)	669	(16)	283	(15)	72	(12)
2	2092	(16)	961	(16)	711	(17)	313	(17)	107	(18)
3 +	3730	(29)	1397	(23)	1284	(31)	767	(41)	282	(46)
Drug comorbidity index										
< Quartile 1	3198	(25)	2404	(39)	635	(15)	140	(8)	19	(3)
Quartile 1–2	3198	(25)	1745	(28)	1105	(26)	297	(16)	51	(8)
Quartile 2–3	3198	(25)	1299	(21)	1225	(29)	529	(29)	145	(24)
> Quartile 4	3198	(25)	700	(11)	1216	(29)	889	(48)	393	(65)
Multi-dimensional diagnosis-based comorbidity index										
< Quartile 1	3198	(25)	2200	(36)	782	(19)	188	(10)	28	(5)
Quartile 1–2	3198	(25)	1830	(30)	1006	(24)	300	(16)	62	(10)
Quartile 2–3	3198	(25)	1383	(22)	1156	(28)	504	(27)	155	(25)
> Quartile 4	3198	(25)	735	(12)	1237	(30)	863	(47)	363	(60)

IQR, inter quartile range (first quartile – third quartile).

The majority of ECOG-PS registrations indicated favourable performance status with 48% (6184) ECOG-PS 0 and 33% (4181) ECOG-PS 1. There were no missing data for age, CCI, MDCI and DCI at date of health care contact, and all four variables increased in parallel with increasing ECOG-PS (Table 1).

Contacts excluded from the study (not made in person or via phone or ECOG-PS not registered on the day of the contact) but with registered ECOG-PS (41 329) were more often performed during 2014–2016, and more often had lower ECOG-PS, e.g. 25 324 (61%) ECOG-PS 0, were slightly younger (median age 74 [IQR: 69–79]), and had similar burden of comorbidity to those with ECOG-PS registered at the date of the health care contact (Supplementary Table 2). This pattern remained when comparing included and excluded contacts within subgroups of ECOG-PS. Excluded contacts without registered ECOG-PS (31 617) were older but had slightly lower comorbidity burden compared to included contacts with ECOG-PS 0.

### Ability to substitute ECOG-PS

**Fig. 1:**
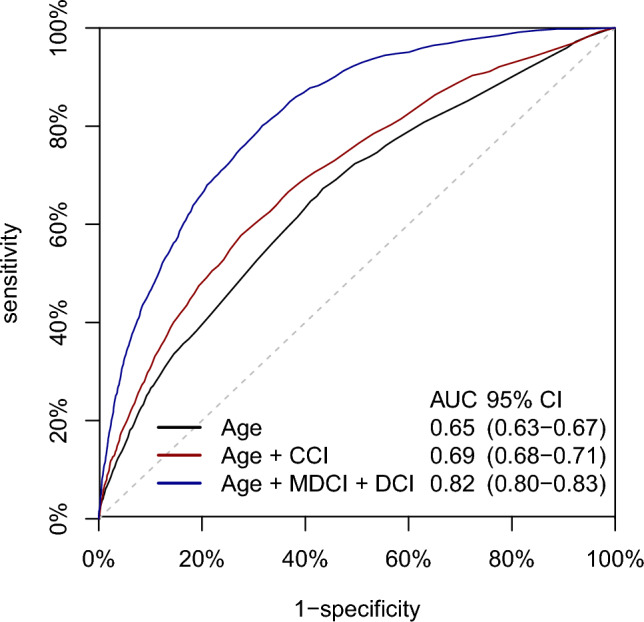
Sensitivity and 1-specificity of the predicted probabilities from modelling the outcome ECOG-PS 0–1 (positive test) versus 2–4 (negative test). AUC, area under the curve; CI, confidence interval.

The ability to discriminate between favourable (N = 10 329 contacts) and unfavourable ECOG-PS (N = 2463 contacts) increased with the number of predictors included in the models. AUC increased from 0.65 (95% CI: 0.63–0.67) for the model including age only, to 0.82 (95% CI 0.81–0.84) for the model including age, MDCI and DCI (Fig. 1). At a specificity of 0.75, the latter model had a sensitivity of 0.72 (95% CI 0.69–0.75), PPV of 0.41 (95% CI 0.39–0.43) and NPV of 0.92 (95% CI 0.91–0.93) (Table 2). At the same specificity, the model including only age had a sensitivity of 0.41 (95% CI 0.35–0.49), PPV of 0.31 (95% CI 0.29–0.34) and NPV of 0.85 (95% CI 0.84–0.86), and the model including age and CCI had a sensitivity of 0.53 (95% CI 0.40–0.57), PPV of 0.34 (95% CI 0.32–0.37) and NPV of 0.87 (95% CI 0.86–0.88) (Supplementary Table 3).

**Table 2:** Sensitivity, and positive and negative predictive values for a given specificity extracted for the model predicting ECOG-PS 0–1 (positive outcome) versus 2–4 (negative outcome) including age, MDCI and DCI. Study prevalence (80%) of ECOG-PS (0–1) was used to compute the PPV and NPV.

Specificity	Sensitivity		Positive predictive value (PPV)		Negative predictive value (NPV)	
	Estimate	95% CI	Estimate	95% CI	Estimate	95% CI
0.5	0.92	(0.90–0.94)	0.31	(0.29–0.33)	0.96	(0.96–0.97)
0.75	0.72	(0.69–0.75)	0.41	(0.39–0.43)	0.92	(0.91–0.93)
0.9	0.46	(0.43–0.50)	0.53	(0.51–0.56)	0.88	(0.87–0.89)
0.95	0.32	(0.29–0.36)	0.61	(0.58–0.64)	0.85	(0.84–0.87)
0.975	0.20	(0.17–0.24)	0.66	(0.63–0.70)	0.84	(0.83–0.85)
0.99	0.10	(0.08–0.13)	0.72	(0.66–0.77)	0.82	(0.81–0.83)

The models provided similar results in the complementary analyses where a favourable performance status instead was defined as ECOG-PS = 0 or ECOG-PS = 0–2 (Supplementary Table 3). Using age, MDCI and DCI, the AUC was 0.81 (95% CI 0.80–0.82) for predicting ECOG-PS 0 versus 1–4 and 0.84 (95% CI 0.83–0.86) for predicting ECOG-PS 0–2 versus 3–4 (Supplementary Fig. 2). For each of the three outcome definitions, the models including only age and one of either MDCI or DCI performed comparably or slightly worse that the model including all of age, MDCI and DCI, while the addition of CCI to a model including MDCI, DCI or both, did not materially change the AUC.

Calibration was similar for all models for all three outcome definitions, with absolute differences between predicted probabilities and empirical averages within 0.01, and brier scores were generally somewhat lower when more variables were included in the model (Supplementary Fig. 3).

The ROC curves and AUC did not materially change when also health care contacts with a retrospectively registered ECOG-PS were included in the analysis (Supplementary Fig. 4).

### Implications for target trial emulation

We evaluated a theoretical example where the inclusion criteria ECOG-PS 0–1 is to be substituted and applied to 1000 men, where 75% have favourable ECOG-PS. Our model based on age, MDCI and DCI and a cut-off to specificity of 0.75 would on average select 600 (60%) men to the study (Table 3). Of these, 10% (60 men) would actually have an unfavourable ECOG-PS.

**Table 3:** Sample size in a population of 1000 men with a fixed hypothetical proportion (prevalence) of ECOG-PS (0–1) when the model is used to select men with ECOG-PS 0–1 to the study population and exclude men with ECOG-PS 2–4, depending on chosen specificity.

Assumed proportion (%) of the population with true ECOG-PS 0–1	Performance		Sample size selected by the model		Included withunfavourable ECOG-PS	
	Specificity	Sensitivity	N	%	N	%
25	0.75	0.72	370	37	189	51
	0.9	0.46	190	19	74	39
	0.95	0.33	120	12	37	31
	0.99	0.10	30	3	7	23
50	0.75	0.72	480	48	125	26
	0.9	0.46	280	28	50	18
	0.95	0.33	190	19	25	13
	0.99	0.10	60	6	5	9
75	0.75	0.72	600	60	60	10
	0.9	0.46	370	37	26	7
	0.95	0.33	260	26	13	5
	0.99	0.10	80	8	2	3
95	0.75	0.72	700	70	14	2
	0.9	0.46	440	44	4	1
	0.95	0.33	320	32	3	1
	0.99	0.10	100	10	1	1

## Discussion

### Summary of findings

Two new comorbidity indices based on comprehensive information from health care registers and age substituted ECOG performance status with reasonable precision for use as proxies for cohort selection when ECOG-PS is unavailable.

### Strengths and limitations

NPCR captures virtually all incident cases (> 98%) with prostate cancer in Sweden compared to the Cancer Register to which registration is mandated by law. The ECOG-PS measurements used in this study were registered in a study population identified in the Patient-overview Prostate Cancer (PPC) during routine clinical care. All centres contribute data in a standardised way through the same platform (INCA; the Information Network for Cancer Care).

We had access to comprehensive high-quality data on comorbidity through linkage with population-based health care registries including the Patient Register and the Prescribed Drug Register^10,16,35^. The version of the CCI used in our study has been specifically adapted to the Swedish Patient Register^36^. The CCI is therefore optimized to the data source. Comorbidity was also measured by use of two new indices (MDCI and DCI) that both have been shown to predict mortality better than the CCI^22,23,37^.

Our study cohort did not include all men with advanced prostate cancer on ADT in Sweden, which is a potential limitation. Previous studies have not raised any major concerns that data in PPC is not representative of men with prostate cancer in Sweden^15,38^. In the main analysis we only used data on ECOG-PS that had been recorded on the day of the health care contact since we anticipated a priori that retrospectively recorded data could be of poorer quality. However, comorbidity burden was comparable between the two groups within each level of ECOG-PS and performance of the models was similar to the main analysis when also contacts with retrospectively recorded ECOG-PS were included, which is a strength.

The underlying purpose of ECOG-PS is to accurately reflect the patient’s performance status, but the assessment of performance status may however not be straight forward^39^. It is a subjective measure susceptible to inter- and intra-rater variability^40–43^ that may fail to account for multimorbidity, frailty and cognitive function. Female patients may also be scored higher than male patients^44^. ECOG-PS is still a commonly used measure of performance status frequently used for selection to oncological RCTs^1–7^. Although widely used in clinical practice, the ECOG-PS is an inherently subjective measure, relying on the judgment of individual healthcare providers. This subjectivity, combined with inter-rater variability, may lead to misclassification of ECOG-PS, thereby weakening the associations between ECOG-PS and key predictors such as age and comorbidity. Such misclassification can compromise the internal validity of our model and diminish its predictive performance when benchmarked against ECOG-PS. Conversely, for research purposes, models based on comorbidity indices derived from healthcare registers may mitigate bias arising from subjectivity and inter-rater variability, offering more consistent predictions of treatment tolerance and survival.

The study population consisted of men with advanced prostate cancer and the utility of our models in women can therefore not be determined from our study. Linkages of rich data sources at an individual level can only be done in some countries. This may limit the use of this method in other settings. Associations between ICD-codes and ATC-codes and ECOG-PS may also be context-sensitive because disease patterns, coding practices, and assessment of performance status are likely to differ between countries and over time. External validation is therefore needed in other settings.

### Implications for observational studies

The patient’s physical performance status is rarely available in data sources used for epidemiological studies. Particularly for target trial emulations, proxy measures for ECOG-PS are needed. Our results support that optimized comorbidity indices can be used for selecting patients with favourable ECOG-PS in observational register-based studies. Using these types of models for approximation, probability cut-offs that balances sensitivity and specificity appropriately must be selected. High specificity is desirable but reduces the available sample size.

The models in our study were developed for use in register-based non-interventional studies and not for application on individual patients in clinical practice. Future research should assess the possibility of substituting ECOG-PS in a general population, including both men and women.

The comorbidity measures, and in particular the MDCI and DCI, take values that are difficult to interpret and may therefore be less suitable for patient selection than ECOG-PS. The MDCI and DCI have been shown to be better surrogates for overall health than the CCI in men with prostate cancer and to improve confounding adjustment in a comparative study of treatment effectiveness^37,45^. The MDCI and DCI may potentially also be better reflecting overall health and improve adjustment for confounding than ECOG-PS, but this is also an open research question that is out of scope of the current study.

### Other studies

In a previous study of 4488 patients with lung cancer a model based on CCI, number of prescribed drugs in the previous year, and other variables, was used to discriminate ECOG-PS 0–2 from 3–5. AUCs of 0.76 and 0.73 were reached in development and validation cohorts, respectively^13^. In another study, models discriminating ECOG-PS 0–1 versus 2–4 were developed for patients with advanced non-small cell lung cancer, advanced bladder cancer, or advanced melanoma, using CCI, sociodemographic, clinical, and laboratory measures^14^. AUCs ranged between 0.71 to 0.80. In our study MDCI and DCI outperformed CCI.

## Conclusion

Using two new comorbidity indices based on comprehensive information from health care registers and age, we were able to discriminate favourable performance status more accurately than when using the Charlson Comorbidity Index. ECOG performance status is often used to select patients to oncological clinical trials, but ECOG-PS is rarely available health care registers. To emulate target trials using administrative health care registers and clinical cancer registers lacking ECOG-PS, these new comorbidity indices can help identify subsets of study populations likely to have favourable ECOG-PS.

## Supplementary Information

Supplementary Information 1.

Supplementary Information 2.

Supplementary Information 3.

## Data Availability

Data used in the present study was extracted from the Prostate Cancer Database Sweden (PCBase), which is based on the National Prostate Cancer Register (NPCR) of Sweden and linkage to several national health-data registers. The data cannot be shared publicly because the individual-level data contain potentially identifying and sensitive patient information and cannot be published due to legislation and ethical approval (https://etikprovningsmyndigheten.se). Use of the data from national health-data registers is further restricted by the Swedish Board of Health and Welfare (https://www.socialstyrelsen.se/en/) and Statistics Sweden (https://www.scb.se/en/) which are Government Agencies providing access to the linked healthcare registers. The data will be shared on reasonable request in an application made to any of the steering groups of NPCR and PCBase (contact npcr@npcr.se). To request data or analytic code from this study, contact the corresponding author. For detailed information, please see www.npcr.se/in-english, where registration forms, manuals, and annual reports from NPCR are available alongside a full list of publications from PCBase.

## Abbreviations

ECOG-PS: Eastern cooperative oncology group performance status
CCI: Charlson comorbidity index
DCI: Drug comorbidity index
MDCI: Multidimensional diagnosis-based comorbidity index
PCBase: Prostate cancer data base Sweden
NPCR: National prostate cancer register
PPC: Patient-overview prostate cancer
ROC: Receiver operating characteristic
AUC: Area under the curve
